# Rule of Law, Corruption Control, Governance, and Economic Growth in Managing Renewable and Nonrenewable Energy Consumption in South Asia

**DOI:** 10.3390/ijerph182010637

**Published:** 2021-10-11

**Authors:** Haider Mahmood, Muhammad Tanveer, Maham Furqan

**Affiliations:** 1 Department of Finance, College of Business Administration, Prince Sattam Bin Abdulaziz University, Alkharj 11942, Saudi Arabia; 2 Prince Sultan University, Riyadh 11586, Saudi Arabia; mtanveer@psu.edu.sa; 3 Oregon State University, Corvallis, OR 97331, USA; furqanm@oregonstate.edu

**Keywords:** governance indicators, renewable and nonrenewable energy consumption, economic growth, South Asia

## Abstract

Strong governance is vital for developing environmental policies to promote renewable energy consumption and discourage nonrenewable energy sources. The present research explores the effect of economic growth and different governance indicators on renewable and nonrenewable energy consumption in Pakistan, India, Bangladesh, and Sri Lanka using data from 1996 to 2019. For this purpose, the study uses different econometric techniques to find the long-term effects of the rule of law, regulatory quality, corruption control, government effectiveness, political stability, voice and accountability, and economic growth on oil, natural gas, coal, hydroelectricity, and renewable energy consumption. The results show that economic growth has a positive impact on all investigated renewable and nonrenewable energy sources. Additionally, regulatory quality measures also increase all types of renewable and nonrenewable energy consumption. Except for natural gas, the impact of the rule of law is negative, and government effectiveness positively affects all energy sources. Control of corruption has a positive effect on natural gas consumption. Political stability has a negative effect on nonrenewable energy sources and a positive impact on renewable energy sources. The magnitudes of the effects of economic growth and most governance indicators are found to be larger on nonrenewable sources than renewable sources. The testing of the energy consumption and governance nexus is scant in global literature and is missing in South Asian literature. Hence, the study results contribute to how South Asian economies can be more sustainable in energy use by enhancing governance indicators in the economies. Particularly, the results imply that these countries should focus on improving the rule of law, corruption control, governance, regulatory quality, political stability, and economic growth to help maintain a sustainable balance of renewable and nonrenewable energy sources. Moreover, this issue needs further attention in developing countries, as governance indicators would play an effective role in promoting sustainable energy.

## 1. Introduction

According to the Global Climate Risk Index 2019, South Asia is one of the most vulnerable regions in the world for risks of climate change as it has extreme weather conditions, including hurricanes, storms, rainfalls, and floods, which affect hundreds of lives every year. In 2017, in India alone, 2726 people lost their lives to extreme weather conditions [1]. These natural disasters are raising the global community’s concerns to slow down the aggressive consequences of climate change across the world. One of these efforts is the Paris Agreement to reduce global warming by 1.5 °C, and the Global Climate Action Tracker to rank countries in policy efforts towards slowing down global warming. South Asia accounts explicitly for one-fourth of the worldwide population, where many people live below the poverty line and struggle to access life necessities, including food, clean water, and shelter [2]. Pakistan, India, Bangladesh, and Sri Lanka are vulnerable communities, e.g., 100 million people are at risk of a climate-related disaster in Pakistan. By 2050, over 40 million people in India will be at risk of a drastic sea-level rise. People in these countries have challenges accessing clean drinking water, and the per capita carbon emissions rate is about 1.4 metric tons [3]. In the midst of all these challenges, it can be hard to keep track of renewable and nonrenewable production and maintain a good pace towards meeting annual emission reduction or zero net emissions goals to complete global climate negotiations.

Pakistan, India, Bangladesh, and Sri Lanka are in a geographically unique region on the globe, which provides them with an abundance of natural resources, including natural and renewable sources to generate power. Pakistan produces 457 kWh per capita electricity, and this number for India, Bangladesh, and Sri Lanka is 644, 278.1, and 636.3, respectively [4]. However, it is essential to note that, in these South Asian countries, access to electricity has still not reached 100%. In Pakistan, 91% of people have access to electricity, and only 36% have access to non-solid fuel. For India, these percentages are 75% and 42%, respectively. In Sri Lanka, 85% of people have access to electricity, but only 25% have access to non-solid fuels, and in Bangladesh, these numbers are much lower, at 55% and 9%, respectively [4]. It should be noted that South Asia is also one of the fastest-growing regions in the world in many domains. The income of this region is three times larger than the income of the year 2005. Overall, the regional economy is growing at a rate of more than 4%, and the poverty rate has fallen to 14% from 47% in 1990. The population of this region is expected to rise by 25% by 2050, which would mean a large jump in resource demand, including energy. By 2025, energy demand in the power sector in South Asia will increase by 93%, and the industry sector will be on the second spot with a 44% increase in energy demand [5]. South Asia is making efforts to increase renewable energy consumption (REC) contributions to achieving global warming reduction targets. Still, nonrenewable consumption is very high in South Asian countries. Table 1 shows the trends of the five-year average of the percentage of energy sources in total energy consumption in the study’s sample period and the average of all years in the sample period. The use of renewable energy was rising slowly in all investigated countries in the sample period from 1996 to 2019. However, the percentage of the use of nonrenewable was found to be 98.9%, 93.3%, 88.7%, and 81.3% in Bangladesh, India, Pakistan, and Sri Lanka, respectively.

There are various policy challenges present, including lack of incentives, large subsidies provided to fossil fuel users, poor environmental regulatory structure, higher costs, complicated tariff structure, lack of technology and expertise, small economies of scale, market uncertainties, and many more [4]. Moreover, it is crucial to understand that many policy-related aspects can affect the industrial and economic transition in a positive or negative way. Nevertheless, energy policymaking or determining a country’s energy mix to control emission levels can be a reasonably political domain that can be influenced by the rule of law, corruption, governance, and economic growth. For instance, Muhammad and Long [6] mentioned in their paper that corruption control, governance, and the rule of law could help keep pollution emissions at a lower level and can help improve environmental quality. Likewise, Mahmood and Alanzi [7] argued that rule of law tends to improve the environment by reducing emissions.

**Table 1:** Five-year average of the percentage of energy sources in total energy consumption.

	Years	Oil	Natural Gas	Coal	Hydroelectricity	REC
Bangladesh	1996–1999	33.5232	63.0850	1.5745	1.8170	0.0002
	2015–2019	23.2853	69.4784	6.4942	0.5749	0.1672
	1996–2019	25.3619	69.4914	4.0476	1.0411	0.0581
India	1996–1999	33.0070	6.7452	53.6225	6.4342	0.1910
	2015–2019	29.7417	6.2553	56.8293	4.0608	3.1129
	1996–2019	31.3887	7.1544	54.7949	5.2299	1.4322
Pakistan	1996–1999	46.2157	33.7363	5.5875	13.8805	0.5800
	2015–2019	33.5880	45.4903	10.7689	9.1619	0.9909
	1996–2019	36.8269	44.4510	7.3939	10.9047	0.4234
Sri Lanka	1996–1999	74.9124	0.0000	0.0058	25.0579	0.0239
	2015–2019	66.8935	0.0000	17.2286	14.1669	1.7110
	1996–2019	75.9532	0.0000	5.3762	18.1370	0.5336

Source: BP [8].

Chang and Li [9] argued that, due to the increasing population and, hence, growing energy demands, South Asian countries are not only using their nonrenewable resources, but they are also importing nonrenewable sources, and this trend seems to be sustained. Another reason that contributes to more use of nonrenewable energy than renewable energy in this part of the world is the poor infrastructure to conserve energy. Since renewable energy sources have specific cycles and mostly require battery storage or other mechanisms to save excess energy, many countries cannot afford the infrastructure. Therefore, the conventional methods of energy production, primarily nonrenewable, seems to be a better option for them. This point leads to the discussion on how governance can set the trend for nonrenewable and renewable energy. Understanding this dynamic would make it easier to comprehend if governance in South Asian countries could promote or hinder any specific energy sources and would provide a pathway to suggest an alternate policy implication. 

With the energy sector making headway towards renewable energy and upgrading energy policymaking on an ongoing basis, the discussion on energy consumption is not just limited to the availability of natural resources and available infrastructure anymore. Countries that are ahead of the game are focusing on institutional and social aspects of their economies and exploring ways of improving energy consumption patterns and making them more sustainable. With rising climate change and global warming concerns, every single attempt would count to make the energy sector more sustainable, no matter how trivial it might sound. Besides, institutional and governance factors like the rule of law, control of corruption, regulatory quality, government effectiveness, political stability, and voice and accountability would play an essential role in helping streamline the energy sector. The rule of law would ensure confidence in law and the legal institution of the country. Control of corruption shows the public power to control all types of corruption in the countries. Regulatory quality depicts the ability of the government to frame regulations to promote private sector development. Government effectiveness makes sure that public services and policies are independent of political pressure. Violence and accountability reflect the freedom of expression, association, and media. Lastly, political stability shows the absence of political instability and terrorism in the countries. South Asian countries like Pakistan, India, Bangladesh, and Sri Lanka have constantly struggled with institutional quality. However, corruption and poor governance are common issues. Therefore, it is crucial to study how these factors contribute to these countries’ energy profiles and devise policies to improve their carbon footprint.

While many recent studies analyze different aspects of the energy policy framework in the South Asian region [10,11,12], the relationship between governance indicators and renewable and nonrenewable energy is somewhat ignored in the context of South Asia. Recent literature has highlighted the role of governance indicators in determining energy consumption [13,14,15,16,17,18]. A lot of the literature is available on developed economies, and there is a considerable gap in the literature when it comes to the South Asian region. This study attempts to bridge that gap. Therefore, it seems pertinent to inquire about the nexus between governance indicators and energy proxies in developing economies, because governance is the most neglected area of any developing country. Hence, the purpose of this paper is to investigate the role of governance indicators that can contribute to renewable and nonrenewable energy use in Pakistan, India, Bangladesh, and Sri Lanka. To achieve our objectives, we apply the Pedroni, Fisher–Johansen, and Westerlund tests for cointegration analyses and used fully modified OLS and dynamic OLS to find the long-term effects of the rule of law, regulatory quality, corruption control, government effectiveness, political stability, voice and accountability, and economic growth on the oil, natural gas, coal, hydroelectricity, and renewable energy consumption. These four countries were selected based on their significant energy consumption levels, higher emissions, population, and economic size. These factors provide these countries with a unique position in the region and make them critical energy players in the energy sector. The results of the study should help to provide more in-depth policy implications for Pakistan, India, Bangladesh, and Sri Lanka on how they can implement broader political frameworks and policies, which can serve the energy sector well and help to maintain a good balance between renewable and nonrenewable sources so that these countries can meet international global warming reduction goals under the Paris Agreement and other commitments. Studying these social, political, and institutional aspects is essential since these countries have rising populations and growing economies. With time, their energy demand is expected to go up. With that rise in energy demand, there is a need to explore better ways to balance renewable and nonrenewable energy so that the increasing energy demand and consumption do not occur at the cost of environmental quality.

The paper is divided into five sections. After this first section, the second section reviews the available literature on the topic and similar aspects. The third section reflects a more narrowed-down theoretical framework and details the methodology, including the data, model, and estimation methods. The fourth section provides an analysis and discussion, while the fifth section delivers concluding remarks.

## 2. Literature Review

Multiple studies talk about the role of governance in energy consumption, energy efficiency, and overall energy policy. For instance, Oberthur et al. [18] provided a global governance context for decarbonization and renewable energy adoption. The study specifically applied the context to energy-intensive industries, including cement, chemical, aluminum, steel, etc. However, using global governance to decarbonize these energy-intensive industries is still underexplored and underutilized. To resolve this issue, the authors suggested forming a centralized institution that can enforce energy policies and help industries maintain a certain net emission goal. This role of centralized governance in the energy sector can also help illuminate how various political aspects, including corruption control, governance, and the rule of law, can impact renewable and nonrenewable energy use in industries. In their research, Lu et al. [16] mentioned that political governance seems to affect energy efficiency profoundly, and so does the corruption perception index. Using some proxies for political governance, the results showed that all proxies improve energy efficiency. Therefore, it is worth exploring the context of energy efficiency and to what extent it supports the use of renewable energy. Cabeca et al. [19] suggested that the European Union needs to improve the overall governance mechanism to improve energy efficiency in the region. Moreover, good governance can also improve access to energy for citizens in a country [20].

According to Komandantova et al. [21], polycentric policies and governance structures in the energy sector can help improve the overall perception and acceptance of people about new energy technology that can improve the overall efficiency of the sector. In addition, Brisbois [22] pointed out that some level of decentralized responsibility is crucial to ensure efficiency in electricity governance. Moreover, Tzankova [23] proposed a combination of the public-private energy sector for renewable energy governance and policy purposes. It indicates that policymaking in the energy sector and well-monitored and well-regulated business operations can significantly determine the right combination of renewable and nonrenewable energy use. In other words, governance has a dynamic and multi-faceted role in deciding the energy mix of a country. In contrast, other strategic and political factors can play their part as well. Hence, corruption control, the rule of law, and political stability would also play a role in using renewable and nonrenewable energy sources. 

Zhang et al. [17] mentioned that renewable energy use is a demand and supply-side phenomenon, and corporate governance and the external rule of law can significantly determine the energy market. The results provided interesting insights into how corporate governance can impact renewable energy adoption. The study analyzed 47 countries and concluded that governance in the energy sector could influence the renewable energy adoption process. For example, board duality was seen to impact renewable adoption negatively, and for companies in common law, this adoption was also relatively lower. Lazaro et al. [24] formulated intriguing qualitative research to analyze the policy and governance dynamics using a case study of biofuels in Brazil. The authors found that governance helped to support the best policies for biofuel management. Putnam and Brown [25] suggested financial mechanisms and community governance for retrofit loans and fuel poverty programs to enhance environmental justice in a country, improve the energy equity situation, and help build a better renewable energy adoption system. While many of these studies talk about the role of governance in the energy sector and directing renewable and nonrenewable energy consumption patterns, there is a need to expand this discussion and make it more inclusive in terms of talking about other political and social instruments, including the rule of law, corruption control, political stability, etc. With only governance, it is hard to predict the role of the political structure of a country in shaping the energy sector, and a more holistic view is necessary.

Caprotti et al. [26] argued that governance should be treated in a multiscale context in the energy landscape because energy transition is a spatialized process. The literature mentioned that energy and the environment have spatial dimensions because pollution is a global phenomenon [27]. While talking about the energy sector transition, political systems, and governance, Alkon and Wong [28] mentioned that decentralization could improve economic prospects for a country. However, it can hinder the performance of the environmental governance structure, and tension between centralized and decentralized political systems can result in cyclicity in the energy system governance. The study also focused on the role of inter-governmental relationships in the context of environmental policies. This discussion can be proved to be crucial in the context of South Asian countries. Although these countries are democratic in general, a combination of centralized and decentralized socio-economic structures and policy frameworks in the energy sector can significantly impact renewable energy adoption, by determining the right energy mix for the countries and devising appropriate energy policies. Moreover, recognizing the social acceptance aspect would help to promote renewable projects [29].

Sanderink [30] pointed to a fundamental flaw in governance mechanisms in the global energy sector. It is mentioned that most of these energy institutions are more focused on climate change and energy access, while the idea of energy scarcity is undermined. Therefore, global energy governance institutions must identify and anticipate energy scarcity issues to improve renewable energy adoption and transition systems and transform energy policies. Bellakhal et al. [15] talked about the poor role of governance in the context of renewable energy investment and, hence, harming energy transitions to renewable energy in the MENA region. The results indicate that a well-structured and framed governance system can help the renewable energy adoption process. Nochta and Skelcher [31] provided another link in the mechanism. The authors mentioned that transition management could help improve the governance network in a country that, in turn, results in a better energy transition.

Khan et al. [13] mentioned that institutional quality could promote renewables and help to reduce emissions in a region. Technological advancements can also help to make it easier to use renewable energy sources, while funding renewable energy can boost this market. Shukla et al. [4] suggested in their paper that, with the use of more advanced technologies and information technologies, countries can tend to develop a better infrastructure and monitoring mechanism for the energy sector, which can help promote the use of renewable energy. Sarkodie and Adams [14] mentioned that an improved political system could enable electricity access to people in Sub-Saharan Africa, which provides a glimpse of how better institutions, governance, and the rule of law can improve the overall energy system. Acheampong et al. [32] found that globalization does not affect energy use. However, economic growth and nonrenewable energy prices have a massive effect on renewable energy consumption (REC) in a country.

In the growth and energy nexus, economic growth accelerates the demand for energy due to the scale effect [33]. This is because the economic growth pushes the consumption and production activities in the country, which require energy to consume. Mahmood et al. [34] investigated the effect of economic growth on different types of energy consumption in Egypt and found a cubic impact of economic growth on oil, primary energy, and coal consumption. Hence, economic growth accelerated the nonrenewable energy use in this developing country. In the same way, Li et al. [35] investigated and corroborated the cubic effect of economic growth on oil, coal, and gas usage in China. In a large panel of 113 countries, Luzzati and Orsini [36] investigated the growth and energy relationship. A monotonic positive effect of economic growth was reported on energy consumption in the panel results. Aboagye [37] investigated the impact of economic growth on energy intensity and consumption in Ghana. A positive impact of economic growth was reported on both energy intensity and consumption. Mahmood et al. [38] investigated and corroborated the quadratic effect of economic growth on oil and gas consumption in Middle Eastern countries. Hence, economic growth increases nonrenewable energy consumption at first, and then reduces with a further level of growth. The same findings are also reported in the country-specific estimation in the case of Iran, UAE, and Kuwait. Mozumder and Marathe [39] investigated the growth and electricity consumption relationship in Bangladesh and reported one-way causality from economic growth to electricity consumption.

In the South Asian context, Murshed [10] investigated and found a positive role of intra-regional trade to boost renewable electricity and energy consumption in South Asia. Hence, regional trade integration can be a key to facilitate renewable energy transitions in this part of the world. In another study on renewable energy use, Murshed et al. [12] suggested that trade openness and foreign currency inflows can also promote the use of renewable energy in South Asia. Furthermore, reducing dependency on crude oil can help boost the renewable energy transition and support countries in this region to adopt more renewable energy [11]. Raza et al. [40] investigated the electricity consumption and income relationship in South Asia during 1980–2010 and found a causality from electricity consumption to economic growth. Xue et al. [41] explored the energy, environment, and institutional quality nexus in South Asia. Renewable energy and institutional quality helped to reduce ecological footprints. Hence, better institutional quality could improve the use of renewable energy and sustain the environment. 

The literature has signified the importance of global governance, political governance, and corporate governance, as well as centralized, decentralized, and polycentric governance in determining energy consumption and efficiency [16,17,18,21,22]. Governance is a broad phenomenon ranging from higher-level global governance to lower-level local or corporate governance. Global governance sets the environmental targets, and political governance at the country and the local levels helps to implement policies to achieve these targets. Thus, the focus of our present study is to capture the effect of governance at the country level on renewable and nonrenewable energy consumption. This is because governance the country level would develop the appropriate energy policies and care for their proper implication to make sure there is a balance for a sustainable future. In this regard, the rule of law, corruption control, political stability, voice and accountability, regulatory quality, and government effectiveness are fundamental aspects of the governance at the country level to investigate. One major limitation of past studies is that they only focus on a few political variables [15,16,42] and do not put the rest of the political structure of their sample countries in the context for a holistic view, which is essential to take into account in order to make well thought out energy policies and recommendations. This current study attempts to fill that gap as it includes maximum governance indicators in the model to investigate the matter in more depth to inform energy policymaking in a better way. Another issue is that most past studies are focused on developed countries or the Western world. There is barely any literature that specifically focuses on South Asia in particular. With their evolving political scenarios and high energy demands, as well as their role in world politics and the global economy, studying these institutional variables and their role in energy consumption can provide a new perspective and help transform a significant chunk of the energy sector across the globe. Considering the above discussion, it seems pertinent to investigate the relationship between governance indicators and renewable and nonrenewable energy consumption of developing countries to understand the extent to which governance indicators may play a role in the sustainable transformation of energy use. Particularly, the investigation of the role of the rule of law, corruption control, political stability, voice and accountability, regulatory quality, and government effectiveness on renewable and nonrenewable energy consumption is missing in Pakistan, India, Bangladesh, and Sri Lanka. Hence, this current study plays a significant role in narrowing down the factors related to governance in determining nonrenewable and renewable energy consumption in this part of the world, and this study explores various policy implications that promote a more sustainable environment.

## 3. Methods

There is sparse research available on the role of governance, institutions, and similar political factors in determining renewable and nonrenewable energy consumption in Asia and even other parts of the world. Therefore, the current study provides a unique perspective on how renewable and nonrenewable consumption can be affected by institutional factors in South Asia, i.e., the rule of law, control of corruption, regulatory quality, government effectiveness, political stability, and voice and accountability. These institutional factors and overall governance can help shape energy consumption trends and what can be done to restructure them according to the country’s energy needs and environmental goals. There are multiple reasons why these factors can impact energy generation and consumption in a region. For instance, Khan et al. [13] suggested that institutional quality could promote renewable energy sources and help reduce pollution. Therefore, institutional quality is an important variable to consider. Without formal institutions with policies to support the energy sector, it is impossible to generate and use energy sources to their best potential, and the entire energy infrastructure can collapse. The role of good governance is essential to understand here, because how energy is being generated and what policy frameworks are available to govern the activities in the energy sector can help set the stage for either renewable or nonrenewable energy activities. Moreover, Chang and Li [9] suggested that poor infrastructure, governance, and policies in Asian countries are among the most common reasons behind the use of nonrenewable energy sources. Therefore, good governance can play a significant role in allowing these countries to see which energy mix combination is the best for them to meet their Paris Agreement and other energy commitment goals. 

The role of corruption and the rule of law cannot be ignored in the context of the energy sector. This is because, for instance, if a country has a high level of corruption, even if there are institutions and governance mechanisms to regulate the energy sector, there is a high chance that companies and environmental polluters would get away with their environmentally degrading activities based on nepotism and bribes [43]. Hence, to plan and execute an efficient energy policy, the energy sector must be corruption free to implement sustainable energy policies. With corruption being prevalent in Pakistan, India, Bangladesh, and Sri Lanka, it is crucial to understand the impact of corruption control on the use of renewable and nonrenewable energy sources. Being in a politically and environmentally challenging part of the world, Pakistan, India, Bangladesh, and Sri Lanka are exposed to many issues that can directly or indirectly hinder the performance of their energy sector. Therefore, it is crucial to understand how the eco-political structure in these countries, including governance indicators and economic growth, can affect renewable and nonrenewable energy sources. It is also important because the increasing population of this region is a grave concern. With higher energy demands, these countries need to develop better and more sustainable energy sources to meet their national energy needs while ensuring that their environmental goals are met in a timely and efficient manner. 

The current paper explores the effects of economic growth and governance indicators on different renewable and nonrenewable energy sources in South Asia. For this purpose, we follow Asongu and Odhiambo’s [42] model, which tested the effect of growth proxies, the rule of law, political stability, corruption control, and voice and accountability on REC. Moreover, we extend the scope of the model investigating both renewable and nonrenewable energy consumption, including regulatory quality and government effectiveness in addition to the proxies used by [42]. The empirical models are as follows:

OIL_it_ = f (Y_it_, ROL_it_, COC_it_, RQ_it_, GE_it_, PS_it_, VA_it_) (1)

GAS_it_ = f (Y_it_, ROL_it_, COC_it_, RQ_it_, GE_it_, PS_it_, VA_it_) (2)

COAL_it_ = f (Y_it_, ROL_it_, COC_it_, RQ_it_, GE_it_, PS_it_, VA_it_) (3)

HYDRO_it_ = f (Y_it_, ROL_it_, COC_it_, RQ_it_, GE_it_, PS_it_, VA_it_) (4)

REC_it_ = f (Y_it_, ROL_it_, COC_it_, RQ_it_, GE_it_, PS_it_, VA_it_) (5)

OIL_it_, GAS_it_, COAL_it_, HYDRO_it_, and REC_it_ are oil, natural gas, coal, hydroelectricity, and renewable energy consumption, respectively, measured in exajoules. All data on energy consumption variables were taken from BP [8]. Y_it_ is the gross domestic product per capita in constant thousands of USD. It was sourced from World Bank [44]. ROL_it_, COC_it_, RQ_it_, GE_it_, PS_it_, and VA_it_ are the rule of law, control of corruption, regulatory quality, government effectiveness, political stability, and voice and accountability, respectively. Definitions of the variables are mentioned in Table 2. These are governance indicators and they ranged from −2.5 to 2.5. An increasing index shows better governance in the country. Data on governance indicators were taken from the World Bank [45]. All data ranged from 1996 to 2019 and were collected for Bangladesh, India, Pakistan, and Sri Lanka. The rest of South Asian countries could not be included because the selected energy variable data were unavailable. ROL_it_, COC_it_, RQ_it_, GE_it_, PS_it_, and VA_it_ were interpolated for the missing years of 1997, 1999, and 2001. The study used a limited time sample because data on governance indicators were not available before 1996. Moreover, a limited sample of four South Asian countries was selected because data on all energy proxies were unavailable for the rest of the South Asian countries. Our study hypothesizes that economic growth and governance indicators may have a statistically positive, negative, or insignificant effect on any type of energy consumption, because improving economic growth and governance indicators could positively affect any kind of energy consumption due to the scale effect [32]. Hence, these actors would increase economic activities and raise energy consumption if the country’s proportions of renewable and nonrenewable energy sources are unchanged. On the other hand, these actors may also reduce the use of nonrenewable energy and increase the use of renewable energy because of technique or composition effects [32]. This is because the economic growth and/or governance in the country would shift the dirty technology into cleaner technology or move the pollution-oriented industry into the sustainable sector. Lastly, statistically insignificant effects may also be expected. Therefore, any type of effect of economic growth and governance on energy consumption is expected, and the exact relationship is an empirical question, which the present study explores in the South Asian context.

Equations (1)–(5) may be tested for panel cointegration if the level series are nonstationary. Hence, we utilize the Im-Pesaran-Shin (IPS) test proposed by Im et al. [46], the Levin-Lin-Shin (LLS) test offered by Levin et al. [47], and the Fisher Augmented Dickey Fuller (ADF) test provided by Maddala and Wu [48]. After testing stationarity, we may apply the cointegration tests on Equations (1)–(5). For this purpose, Johansen [49] proposed the Trace and Maximum Eigenvalues, which can be estimated for the individual South Asian countries’ time series models.

(6) $$\Delta y_{t}=a_{0}+a_{1}y_{t-1}+\sum_{i=1}^{n-1}a_{2i}\Delta y_{t-1}+e_{t}$$

(7) $$J_{trace}=-T\sum_{k=r+1}^{N}ln\left(1-\hat{\emptyset_{k}}\right)$$

(8) $$J_{max}=-Tln\left(1-\hat{\emptyset_{r+1}}\right)$$

where $\hat{\emptyset_{k}}$ is a canonical correlation. Then, the cumulative probability of Trace and Maximum Eigenvalues can be estimated using Maddala and Wu’s [48] methodology to conclude the cointegrating vectors in the panel models using the following:

(9) $$w=-2\sum_{i=1}^{N}log_{e}\left(\pi_{i}\right)$$

Pedroni’s [50] panel cointegration is utilized to test the robustness of Johansen’s results. The following can be used to verify the cointegration in Equations (1)–(5):

Within dimensions:

(10) $$T^{2}N^{1.5}Z_{\hat{v}\;N,T}=T^{2}N^{1.5}{\left(\sum_{i=1}^{N}\sum_{t=1}^{T}1/{\hat{L}}_{11i}^{2}{\hat{e}}_{i,t-1}^{2}\right)}^{-1}$$

(11) $$TN^{0.5}Z_{\hat{\rho \;}N,T-1}=T^{2}N^{0.5}\left(\sum_{i=1}^{N}\sum_{t=1}^{T}1/{\hat{L}}_{11i}^{2}{\hat{e}}_{i,t-1}\Delta {\hat{e}}_{i,t}-{\hat{\lambda }}_{i}\right){\left(\sum_{i=1}^{N}\sum_{t=1}^{T}1/{\hat{L}}_{11i}^{2}{\hat{e}}_{i,t-1}^{2}\right)}^{-1}$$

(12) $$Z_{t\;N,T}=\left(\sum_{i=1}^{N}\sum_{t=1}^{T}1/{\hat{L}}_{11i}^{2}{\hat{e}}_{i,t-1}\Delta {\hat{e}}_{i,t}-{\hat{\lambda }}_{i}\right){\tilde{(\sigma }}_{N,T}^{2}\sum_{i=1}^{N}\sum_{t=1}^{T}{\hat{L}}_{11i}^{-2}{\hat{e}}_{i,t-1}^{2}{)}^{-0.5}$$

(13) $$Z_{t\;N,T}^{*}=\left(\sum_{i=1}^{N}\sum_{t=1}^{T}1/{\hat{L}}_{11i}^{2}{\hat{e}}_{i,\;t-1}^{*}\;\Delta {\hat{e}}_{i,t}^{*}\right){\left({\tilde{s}}_{N,T}^{*2}\sum_{i=1}^{N}\sum_{t=1}^{T}1/{\hat{L}}_{11i}^{2}{\hat{e}}_{i,t-1}^{*2}\right)}^{-0.5}$$

Between dimensions:

(14) $$TN^{0.5}{\tilde{Z}}_{\hat{\rho \;}N,T-1}=T.N^{-0.5}\left(\sum_{t=1}^{T}{\hat{e}}_{i,t-1}\Delta {\hat{e}}_{i,t}-{\hat{\lambda }}_{i}\right)\left(\sum_{i=1}^{N}\sum_{t=1}^{T}{\hat{e}}_{i,t-1}^{2}{)}^{-1}\right]$$

(15) $${\tilde{N^{-0.5}Z}}_{t\;N,T}=N^{-0.5}{\left(\sum_{t=1}^{T}{\hat{e}}_{i,t-1}\Delta {\hat{e}}_{i,t}-{\hat{\lambda }}_{i}\right)}^{-1}\sum_{i=1}^{N}{\left({\hat{\sigma }}_{i}^{2}\sum_{t=1}^{T}{\hat{e}}_{i,t-1}^{2}\right)}^{-1}$$

(16) $$N^{-0.5}{\tilde{Z}}_{t\;N,T}^{*}=N^{0.5}{\left(\sum_{t=1}^{T}{\hat{e}}_{i,\;t-1}^{*}\Delta {\hat{e}}_{i,\;t}^{*}\right)}^{-1}.\sum_{i=1}^{N}{\left(\sum_{t=1}^{T}{\hat{s}}_{i}^{*2}{\hat{e}}_{i,t-1}^{*2}\right)}^{-0.5}$$

After testing Equations (10)–(16), we apply Westerlund’s [51] cointegration approach to validate the cointegration in Equations (1)–(5), which removed the restriction of common factors. The test statistics are as follows:

(17) $$G_{T}=N^{-0.5}\sum_{i=1}^{N}{\hat{\alpha }}_{i}/S.E\left({\hat{\alpha }}_{i}\right)$$

(18) $$G_{\alpha }=N^{-0.5}\sum_{i=1}^{\;N}\left(\frac{t{\hat{\alpha }}_{i}}{{\hat{\alpha }}_{i}\left(1\right)}\right)$$

(19) $$P_{T}=\hat{\alpha }/S.E\left(\hat{\alpha }\right)$$

(20) $$P_{\alpha }=t\hat{\alpha }$$

After confirmation of cointegration, we apply the fully modified ordinary least square (FMOLS) of Pedroni [52], which cares about endogeneity and serial correlation. The FMOLS estimators can be calculated as follows:

(21) $${\hat{\beta }}_{FMOLS}=\overset{N}{\underset{n=1}{\sum }}\left(\overset{T}{\underset{t=1}{\sum }}\left(x_{it}-{\bar{x}}_{i}\right){\hat{y}}_{it}^{+}+T{\hat{\Delta }}_{\varepsilon \mu }^{+}\right){\left(\overset{N}{\underset{i=1}{\sum }}\overset{T}{\underset{t=1}{\sum }}{\left(x_{it}-{\bar{x}}_{i}\right)}^{\prime }\right)}^{-1}$$

where ${\hat{\Delta }}_{\varepsilon \mu }^{+}$ and $y_{\varepsilon \mu }^{+}$ remove serial correlation and endogeneity, respectively. Finally, to test the robustness of the FMOLS results, the dynamic ordinary least square (DOLS) of Kao and Chiang [53] is applied, considering the lead and lag in the model in the following way:

(22) $$Y_{it}=c_{i}+X_{it}^{\prime }d+\sum_{n=-q}^{n=+q_{2}}f_{ik}\Delta x_{i,t+n}+e_{it}$$

Moreover, DOLS estimators can be calculated as follow:

(23) $${\hat{d}}_{DOLS}=\left(\sum_{t=1}^{T}x{\hat{x}}_{it}^{+}\right).\;\sum_{i=t}^{N}{\left(\sum_{t=1}^{T}x_{it}x_{it}^{’}\right)}^{-1}$$

## 4. Results and Discussions

First, we tested the unit root in the panel series. Table 3 shows the results of the LLC, IPS, and Fisher-ADF tests. All series have a unit root in their level. However, all series are stationary after first differencing at various levels of significance. So, the order is integration is one in all the hypothesized models. 

Table 4 shows the panel cointegration results of the five models of energy consumption. At first, we discuss the Pedroni test. In the oil model, cointegration is corroborated with four within-dimension statistics, two within-dimension weighted statistics, and two between-dimension statistics. Cointegration is found in natural gas and coal models with one within-dimension statistic, two within-dimension weighted statistics, and two between-dimension statistics. In the hydroelectricity model, cointegration is validated with four within-dimension statistics, three within-dimension weighted statistics, and three between-dimension statistics. In the REC model, cointegration is verified with three within-dimension statistics, two within-dimension weighted statistics, and two between-dimension statistics. In all models, the Fisher–Johansen test provides strong evidence of cointegration with the eight cointegrating vectors in both Maximum Eigen and Trace statistics. Finally, the Westerlund test validates the cointegration with four statistics in the hydroelectricity model and two statistics in the oil, gas, coal, and REC models.

Table 5 shows the results of FMOLS and DOLS, and Table 6 shows the matrix of relationships. Y_it_ has a positive and statistically significant effect on all types of energy sources. Hence, increasing economic growth accelerates the demand for renewable and nonrenewable energy and shows a scale effect on all energy sources [54]. It makes theoretical sense as a country becomes more economically advanced and grows. There is a higher rate of industrial and manufacturing activities and a rapid flow of transport and urbanization, which inevitably increase energy demand [55,56]. Economic growth has the most significant effect on coal consumption, followed by oil consumption. Coal and oil have been primary fuels in these countries, and it is no surprise that they still heavily rely on these fuel types. One of the reasons is that switching to renewables is not as simple as it seems and requires restructuring the grid and relevant infrastructure, which is costly and comes with many governance and regulation issues. 

While renewable is on the rise across the world, there are certain regulatory and infrastructure limitations that even developed economies are facing, which makes a complete integration of renewables into the conventional grid challenging. For developing countries, switching to renewables is even more challenging without the necessary infrastructure and enough research to back up all the industry work. Additionally, coal and other fuel-type industries in these four countries are a huge source of income and provide employment for a large number of people, which is why suddenly removing this industry altogether and transforming it into something completely new is not as feasible. Therefore, these countries stick to the old mechanisms and keep using coal and other fossil fuels. Thus, the growth of South Asia may negatively affect the environment. 

RQ_it_ positively affects all types of energy uses, indicating that regulatory quality promotes private sector activities through improved government policies. Hence, regularity quality may promote economic activities and raise renewable and nonrenewable energy consumption through the scale effect. In the case of renewable energy, the literature has corroborated that governance indicators, political systems, and institutional quality are fundamental to increasing the country’s REC [14,18,19]. This result also provides a potential policy implication for the limitation of the energy sector in South Asia and the inability to suddenly switch to renewables. Better regulatory mechanisms and government policies backing up sustainable energy practices can help improve the overall energy profile in South Asia and make energy consumption more environmentally friendly. Regulatory agencies can also enforce antitrust laws in the energy sector to make sure that there is fair competition in the market and quality service is being provided to the customers, which may improve the overall quality of the energy sector. Moreover, the magnitudes of effects on nonrenewable energy sources are more than those on renewable sources. Hence, this result also shows that government policies are less concerned with promoting renewable consumption in the private sector. Abid [57] argued that strong institutions would promote foreign investment with environmentally friendly technologies, which can have a technical effect in the country to promote renewable energy. Hence, the positive impact of regularity quality on renewable energy usage shows that the awareness of renewable energy is at least increasing with increasing regularity quality in South Asia. However, it might take some time to spread enough awareness in these countries about renewable energy so that the common public starts adopting it and people consider installing rooftop solar panels in their homes. Nevertheless, better regulatory frameworks and institutional quality can certainly be a stepping stone to elevate overall energy practices and expedite the transformation process.

ROL_it_ has a negative effect on all energy sources except natural gas consumption. The rule of law reflects the perception of individuals about the quality of institutions related to law and order in the country and may help implement environmental policies. Furthermore, strong law and order would ensure the application of environmental regulations for fear of accountability [58]. The negative effects of the rule of law on nonrenewable energy are in line with the theoretical predictions that it helps implement environmental policies and discourages nonrenewable energy consumption. The literature has also corroborated that the rule of law helps to transform energy use [15,18]. Moreover, Lu et al. [16] argued that good governance helps to increase energy efficiency. Therefore, it may help to reduce overall energy use in any country. Stricter policies and rule of law are crucial to ensure that governments, manufacturers, big polluters, and regulatory agencies are following international treaties and making their way towards meeting global climate goals. With stricter policies, countries are able to enforce emission taxes and other laws in the energy sector, resulting in more renewable energy penetration and reduced emissions. In this context, Salman et al. [59] reported that a strict rule of law decreased the pollution level. On the other hand, the negative effect of the rule of law on renewable energy shows that South Asian countries have less concern about promoting renewable energy in their legal frameworks. This finding is in line with Abid [60], who found that the rule of law accelerated pollution emission. It may be related to the fact that a massive chunk of these economies depends on nonrenewable energy as the fossil fuel business provides many jobs. Therefore, if these countries make stricter energy laws and regulations, they may halt their economic activities and growth. Consequently, they prefer focusing on economic growth for now while energy policies remain less strict, because improving law and order may reduce economic activities, which would reduce the demand for all sources of energy. 

COC_it_ has a positive effect on natural gas consumption. Control of corruption reflects the effort of public powers to promote the general private interest. Therefore, it was found that improving control of corruption could only accelerate nonrenewable energy. In a counterargument, Arminen and Menegaki [61] argued that corruption is a hurdle in implementing environmental regulation. Hence, improving corruption control should reduce the consumption of nonrenewable energy. On the other hand, Hassan et al. [62] reported that corruption was responsible for environmental degradation in Pakistan. Moreover, Larraín and Tavares [43] argued that economies with weak institutions welcome more foreign investments because foreign investors might bribe the bureaucracy to break the environmental rules. Moreover, Oberthur et al. [18] also corroborated the role of corruption control in determining energy consumption. Hence, our finding of a positive effect of COC_it_ on natural gas reflects that South Asian countries do not have reasonable ecological regulations to reduce the use of nonrenewable energy. Moreover, improving COC_it_ increases economic activities and increases natural gas demand through the scale effect. 

GE_it_ has a positive effect on all types of energy except natural gas. Government effectiveness represents the independence of public services from political pressure and may support economic activities by providing better public services. Hence, government effectiveness promotes all types of energy sources except natural gas. Moreover, the magnitudes of effects of nonrenewable energy sources are multiple times more than renewable sources. In this context, Galinato and Galinato [63] argued that a weak government would be pressurized with lobbies in the countries, which may be a hurdle in the way of renewable energy policies. Hence, public services are not promoting renewable energy sources effectively. However, improving government effectiveness is at least increasing the consumption of renewables. 

PS_it_ has a negative effect on the nonrenewable use of energy and positively impacts renewable energy sources. Hence, our finding reflects that political stability helps to promote renewable energy and discourages nonrenewable energy in South Asia and vice versa. As per the theoretical prediction, this is a relevant result as a long and stable government may generate renewable energy production capacity, which requires installation time. Hence, increasing dependence on renewables would reduce the economy’s dependence on nonrenewable sources. On the other hand, political instability would lead to the government’s weaker position to frame environmentally friendly policies, because the unstable government would be under pressure from local and foreign business lobbies and could not implement strict environmental policies [64]. Lastly, VA_it_ has a statistically insignificant effect on all types of energy uses. Voice and accountability represent the extent of freedom to choose the government as well as press freedom. This result reflects that voice and accountability indicators are fragile in South Asian countries. Hence, they could not affect any type of energy usage. DOLS was applied to test the robustness of the FMOLS results, and the conclusions remain the same. 

## 5. Conclusions

Institutions may play their role in adopting cleaner technologies for a healthier environmental quality. South Asian countries consume more than 80% of nonrenewable energy sources and have a low level of governance. Without good governance, the transformation of the economy from using nonrenewable to renewable energy sources is not possible. Hence, this study examines the impact of economic growth and different governance indicators on renewable and nonrenewable energy sources in South Asia from 1996 to 2019. We found that economic growth accelerated all types of energy usage and has a larger magnitude of effects on nonrenewable energy. Therefore, as these South Asian countries achieve economic growth, the use of nonrenewable energy sources increases faster than the renewable ones, which leads to environmental degradation. These countries have been relying on nonrenewable energy sources for a long time and the nonrenewable energy industries are responsible for the jobs of millions of people. Therefore, without the proper infrastructure, research, and awareness in the public, it is hard to switch to renewable energy and make a seamless structural transition. It explains why nonrenewable energy sources seem to contribute more to the economies of these South Asian countries than renewables. This finding leads to a policy recommendation that, while setting economic growth goals, these countries need to cater to environmental goals at the same scale to ensure that the costs of ecological degradation does not outweigh the benefits of economic growth.

It might be a long process, and they might end up getting some pushback and temporary repercussions. However, it is essential to understand that making the energy sector more sustainable is going to help their economies in the long run. Regulatory quality has positive effects on all energy sources and has stronger effects on nonrenewable than renewable energy sources. Hence, improving regulatory quality promotes overall energy consumption through the scale effect in South Asia. However, regulatory bodies should encourage renewables. With better regulatory policies and stricter policies in general, companies responsible for environmental degradation would have to abide by the laws and regulations in a more structured way and monitor their business activities, which could lead to lessening environmental degradation. Hence, with pollution taxes and other financial penalties, better regulation would help to restrict environmental degradation activities and keep things within the limits of industry and international environmental goals.

The rule of law hurts all energy usage except natural gas consumption. Thus, improving the rule of law could be helpful to reduce overall energy consumption in South Asia. It is so because a stronger rule of law would put more restrictions on the energy sector and limit the use of various energy sources. However, it should be noted that it can help economies in the long term, as regulating energy use may provide more space for improvements in energy infrastructure and innovation to find better ways to produce and consume energy. Moreover, it also shows that environmental regulations are weak to control the use of natural gas. Hence, South Asian countries should focus on improving the rule of law indicators and tracing policies to control nonrenewable energy in the region. These combined efforts would help to reduce the use of nonrenewable energy and to improve the environment. 

Control of corruption positively affects natural gas consumption and has insignificant effects on other renewable and nonrenewable sources. Government effectiveness has a positive impact on all renewable and nonrenewable energy except natural gas and has a greater magnitude of impact on nonrenewable than renewable energy sources. Political stability has a negative effect on nonrenewable energy consumption and positively affects renewable energy consumption. Hence, political stability allows the government to install renewable energy capacity, which needs a long time to be installed and to replace the consumption of nonrenewable energy. Therefore, South Asian countries are suggested to promote political stability indicators to support renewable energy for a sustainable environment. Lastly, voice and accountability did not affect any energy type.

Getting back to the points made in the Introduction and Literature Review Sections, these results help fill in the literature gap and understand how vital political instruments can help the energy sector’s stability. There is no denying that the role of these political factors in the energy sector is underestimated in South Asia, and more research like this study can help to pave the way to more fruitful discussions on the topic that can help transform the South Asian energy sector. Unfortunately, the present study only investigated four South Asian countries and used a limited time sample due to the non-availability of data. However, future research on the topic may extend the research scope by expanding the sample size. Moreover, future studies may also focus on combining country-specific governance with global governance related to energy issues to see whether global governance could play a role in transforming South Asia from primarily using nonrenewable sources of energy to using to renewable sources of energy.

## Figures and Tables

**Table 2:** Definition of variables.

Variable	Definition	Unit of Measurement	Source
OIL_it_	Oil consumption including inland, aviation, and marine usage.	Exajoules	[8]
GAS_it_	Natural gas consumption, including natural gas consumed in gas-to-liquids transformation.	Exajoules	[8]
COAL_it_	Commercial solid coal fuels consumption.	Exajoules	[8]
HYDRO_it_	Hydroelectricity consumption is equivalent to the amount of fuel required to produce reported electricity.	Exajoules	[8]
REC_it_	Renewable energy consumption apart from hydro or biofuels.	Exajoules	[8]
Y_it_	Gross Domestic Production per capita	Constant 2010 USD	[37]
ROL_it_	The rule of law reflects agents’ perception of confidence in the law and the legal institutions of the country.	−2.5 (weak) to 2.5 (strong) governance performance	[38]
COC_it_	Control of corruption reflects a perception about public power to control all types of corruption.	−2.5 (weak) to 2.5 (strong) governance performance	[38]
RQ_it_	Regulatory quality reflects the perception of the ability of the government to frame regulations to promote development.	−2.5 (weak) to 2.5 (strong) governance performance	[38]
GE_it_	Government effectiveness reflects the perception of public services, policies, and the degree of independence from political pressure.	−2.5 (weak) to 2.5 (strong) governance performance	[38]
VA_it_	Violence and accountability reflect the perception of the freedom of expression, association, and media.	−2.5 (weak) to 2.5 (strong) governance performance	[38]
PS_it_	Political stability reflects the perception about the absence of political instability and terrorism.	−2.5 (weak) to 2.5 (strong) governance performance	[38]

**Table 3:** Panel unit root test.

Variable	LLC		IPS		Fisher-ADF	
	Intercept	Intercept and Trend	Intercept	Intercept and Trend	Intercept	Intercept and Trend
**Level**						
OIL_it_	2.2974	1.4281	3.1372	1.1286	2.6549	4.9184
GAS_it_	0.6546	−1.1460	2.1411	−0.6498	2.0905	7.2871
COAL_it_	2.9715	0.4465	4.9715	1.4782	0.0832	3.1301
HYDRO_it_	2.7012	5.5523	1.4853	−0.0675	4.2235	6.7494
REC_it_	7.5208	3.3494	6.2423	5.5168	1.8579	0.3990
Y_it_	4.1285	1.8219	4.9667	2.2701	0.5798	6.6073
ROL_it_	−0.5625	0.0694	−0.5582	−0.2510	9.7773	7.8435
COC_it_	−1.2729	−0.5042	−1.3190	−0.7414	12.9959	12.0304
RQ_it_	−0.3983	0.6525	−1.4766	−0.1345	13.7525	6.8658
GE_it_	1.5777	1.5453	0.5441	0.0510	8.6407	7.3616
VA_it_	0.1047	0.9954	−0.7564	0.5225	8.9794	4.5855
PS_it_	−1.0065	0.4652	−0.1973	1.1906	6.2254	5.3033
**First difference**						
ΔOIL_it_	−2.0445 **	−1.7787 **	−2.9152 **	−2.6591 ***	22.4159 ***	21.3443 ***
ΔGAS_it_	−2.1569 **	−1.7860 **	−3.5779 ***	−3.4234 ***	23.9007 ***	22.4301 ***
ΔCOAL_it_	−2.1247 **	−1.5106 *	−1.6924 **	−1.3063 *	15.4672 **	13.6997 *
ΔHYDRO_it_	−7.7429 ***	−6.4384 ***	−7.2678 ***	−5.8570 ***	57.6225 ***	43.0532 ***
ΔREC_it_	−2.1801 **	−2.6891 ***	−1.9098 **	−2.0865 **	13.6387 *	16.6244 **
ΔY_it_	−1.7209 **	−1.6829 **	−1.6185 **	−2.0929 **	15.4897 **	18.6050 **
ΔROL_it_	−3.2676 ***	−2.0522 **	−4.7789 ***	−4.0339 ***	37.0430 ***	30.0746 ***
ΔCOC_it_	−3.7923 ***	−2.6745 ***	−4.1058 ***	−2.9777 ***	31.9128 ***	22.6968 ***
ΔRQ_it_	−3.2911 ***	−2.1233 **	−3.6351 ***	−2.2831 **	27.8451 ***	18.0305 **
ΔGE_it_	−7.7090 ***	−6.4326 ***	−6.7223 ***	−5.6209 ***	53.0943 ***	41.2440 ***
ΔVA_it_	−4.5233 ***	−4.0326 ***	−3.8218 ***	−2.6953 ***	29.3821 ***	20.6549 ***
ΔPS_it_	−5.1972***	−5.2345***	−4.2033 ***	−3.7775 ***	32.7264 ***	28.1788 ***

Note: *, **, and *** depict stationarity at 10%, 5%, and 1%, respectively.

**Table 4:** Panel cointegration.

	OIL_it_	GAS_it_	COAL_it_	HYDRO_it_	REC_it_
Pedroni Test					
Within-dimension					
Panel-v	1.3289 (0.0919)	−0.1892 (0.5750)	0.4345 (0.3320)	1.4703 (0.0707)	4.6766 (0.0000)
Panel-rho	−1.8575 (0.0316)	0.7048 (0.7595)	−0.1145 (0.4544)	−3.2232 (0.0006)	−1.4575 (0.0725)
Panel-PP	−3.1809 (0.0007)	0.6414 (0.7394)	−0.4903 (0.3119)	−3.2973 (0.0005)	−1.0220 (0.1534)
Panel-ADF	−4.6079 (0.0000)	−4.1568 (0.0000)	−1.3889 (0.0829)	−2.2496 (0.0122)	−4.0647 (0.0000)
Within-dimension weighted					
Panel-v	1.0507 (0.1467)	−0.7506 (0.7735)	1.2164 (0.1130)	−1.1301 (0.8708)	−0.4193 (0.6625)
Panel-rho	−1.0790 (0.1403)	0.4369 (0.6689)	−1.1767 (0.1197)	−6.7867 (0.0000)	−5.5987 (0.0000)
Panel-PP	−1.4876 (0.0684)	−1.9103 (0.0280)	−1.6668 (0.0478)	−6.9253 (0.0000)	−6.7241 (0.0000)
Panel-ADF	−1.7613 (0.0391)	−5.1233 (0.0000)	−1.5903 (0.0559)	−3.8255 (0.0001)	−0.2898 (0.3860)
Between-dimension					
Group-rho	−0.0724 (0.4711)	1.2635 (0.8968)	−0.6190 (0.2680)	−4.8067 (0.0000)	0.9972 (0.8407)
Group-PP	−1.3418 (0.0898)	−2.7036 (0.0034)	−1.6736 (0.0471)	−6.9613 (0.0000)	−2.9759 (0.0015)
Group-ADF	−2.1579 (0.0155)	−4.8132 (0.0000)	−1.3767 (0.0843)	−3.6293 (0.0001)	−1.3454 (0.0962)
Fisher–Johansen Trace Test					
None	167.30 (0.0000)	167.30 (0.0000)	197.32 (0.0000)	136.33 (0.0000)	167.30 (0.0000)
At most 1	184.40 (0.0000)	184.40 (0.0000)	184.40 (0.0000)	61.47 (0.0000)	61.47 (0.0000)
At most 2	249.70 (0.0000)	187.30 (0.0000)	249.70 (0.0000)	249.70 (0.0000)	249.70 (0.0000)
At most 3	187.20 (0.0000)	131.20 (0.0000)	155.90 (0.0000)	161.80 (0.0000)	173.90 (0.0000)
At most 4	101.10 (0.0000)	74.13 (0.0000)	80.70 (0.0000)	85.63 (0.0000)	114.60 (0.0000)
At most 5	52.31 (0.0000)	44.77 (0.0000)	48.56 (0.0000)	56.32 (0.0000)	69.39 (0.0000)
At most 6	31.82 (0.0001)	29.38 (0.0001)	25.80 (0.0011)	35.89 (0.0000)	37.41 (0.0000)
At most 7	18.71 (0.0165)	15.51 (0.0167)	17.01 (0.0300)	15.91 (0.0437)	35.35 (0.0000)
Fisher–Johansen Max-Eigen Test					
None	41.89 (0.0000)	41.89 (0.0000)	50.25 (0.0000)	55.26 (0.0000)	41.89 (0.0000)
At most 1	351.6 (0.0000)	351.6 (0.0000)	351.60 (0.0000)	117.20 (0.0000)	117.20 (0.0000)
At most 2	136.1 (0.0000)	102.1 (0.0000)	136.10 (0.0000)	136.10 (0.0000)	136.10 (0.0000)
At most 3	121.8 (0.0000)	90.19 (0.0000)	101.20 (0.0000)	106.60 (0.0000)	107.70 (0.0000)
At most 4	59.75 (0.0000)	37.29 (0.0000)	40.08 (0.0000)	39.20 (0.0000)	62.95 (0.0000)
At most 5	29.84 (0.0002)	23.27 (0.0007)	32.31 (0.0001)	31.51 (0.0001)	43.57 (0.0000)
At most 6	26.45 (0.0009)	24.02 (0.0005)	21.31 (0.0064)	31.28 (0.0001)	22.03 (0.0049)
At most 7	18.71 (0.0165)	15.51 (0.0167)	17.01 (0.0300)	15.91 (0.0437)	35.35 (0.0000)
Westerlund Test					
Statistic					
Gt	−2.173 (0.189)	−1.732 (0.751)	−4.434 (0.000)	−4.476 (0.000)	−1.600 (0.911)
Ga	−9.593 (0.184)	−4.639 (0.924)	−13.095 (0.103)	−15.538 (0.001)	−6.852 (879)
Pt	−6.633 (0.000)	−8.365 (0.000)	−6.913 (0.000)	−5.936 (0.001)	−6.107 (0.016)
Pa	−16.075 (0.000)	−9.854 (0.000)	−8.738 (0.152)	−12.928 (0.000)	−14.722 (0.013)

Note: ( ) contains *p*-value.

**Table 5:** Regression results.

Variable	OIL_it_	GAS_it_	COAL_it_	HYDRO_it_	REC_it_
FMOLS					
Y_it_	0.9741 (0.0006)	0.4930 (0.0000)	1.8951 (0.0054)	0.9521 (0.0043)	0.1771 (0.0032)
RQ_it_	2.7973 (0.0044)	0.5696 (0.0338)	5.5554 (0.0188)	0.2351 (0.0411)	0.5360 (0.0099)
ROL_it_	−3.3313 (0.0020)	−0.4687 (0.1072)	−8.3696 (0.0014)	−0.4201 (0.0010)	−0.5677 (0.0122)
COC_it_	0.5552 (0.5241)	0.5108 (0.0360)	1.0811 (0.6089)	0.0601 (0.5608)	0.1064 (0.5656)
GE_it_	2.7137 (0.0226)	0.4278 (0.1881)	5.9955 (0.0373)	0.3315 (0.0189)	0.6258 (0.0136)
PS_it_	−0.8624 (0.0046)	−0.5263 (0.0000)	−1.7696 (0.0158)	0.1089 (0.0026)	0.1514 (0.0183)
VA_it_	0.3355 (0.6144)	−0.0110 (0.9523)	0.9866 (0.5414)	0.0845 (0.2859)	0.0347 (0.8065)
DOLS					
Y_it_	0.5945 (0.0252)	0.3214 (0.0003)	0.9312 (0.0146)	0.5371 (0.0167)	0.1335 (0.0415)
RQ_it_	1.4446 (0.0744)	0.1486 (0.0535)	2.0634 (0.0292)	0.0553 (0.0641)	0.3229 (0.0104)
ROL_it_	−1.6398 (0.0160)	−0.5399 (0.1242)	−4.7797 (0.0954)	−0.2799 (0.0981)	−0.2269 (0.0427)
COC_it_	0.1647 (0.8111)	0.2303 (0.0268)	0.0339 (0.9839)	0.0262 (0.7986)	0.0554 (0.7445)
GE_it_	2.6375 (0.0247)	0.5143 (0.1393)	5.7927 (0.0428)	0.3182 (0.0665)	0.5745 (0.0463)
PS_it_	−0.5818 (0.0233)	−0.4216 (0.0000)	−1.1051 (0.0753)	0.0838 (0.0279)	0.1078 (0.0851)
VA_it_	0.0225 (0.9735)	0.2407 (0.2391)	0.4793 (0.7722)	0.0887 (0.3800)	−0.0874 (0.6007)

( ) contains *p*-value.

**Table 6:** Matrix of the relationship.

Variable	OIL_it_	GAS_it_	COAL_it_	HYDRO_it_	REC_it_
Y_it_	+	+	+	+	+
RQ_it_	+	+	+	+	+
ROL_it_	−	0	−	−	−
COC_it_	0	+	0	0	0
GE_it_	+	0	+	+	+
PS_it_	−	−	−	+	+
VA_it_	0	0	0	0	0

## Data Availability

The data are publicly available [36,37,38].
